# Supplementary material for: Establishment of a fluorescent reporter of RNA-polymerase II activity to identify dormant cells
Source: Nat Commun. 2021 Jun 3;12:3318. doi: 10.1038/s41467-021-23580-4 (PMC8175728; doi:10.1038/s41467-021-23580-4)
Supplement: Supplementary file 1 — Supplementary Information [file 41467_2021_23580_MOESM1_ESM.pdf]

Supplementary information

Supplementary information includes 6 Supplementary Figures, 2 Supplementary Tables and 1 Supplementary movie.

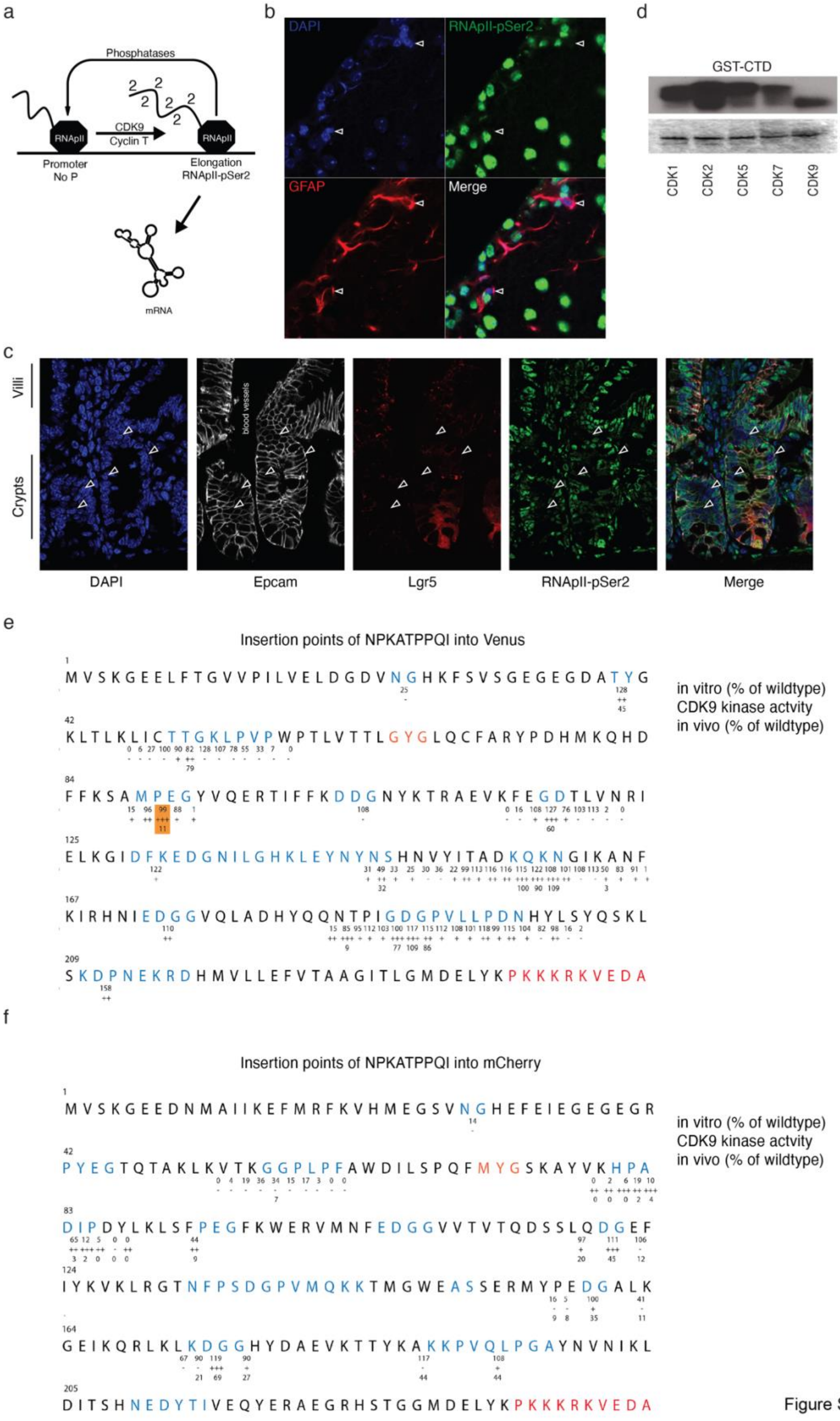
a
Phosphatases
CDK9
Cyclin T
RNApII
Promoter
No P
Elongation
RNApII-pSer2
mRNA
b
DAPI
RNApII-pSer2
GFAP
Merge
d
GST-CTD
32P-γATP kinase assay
Coomassie
CDK1
CDK2
CDK5
CDK7
CDK9
c
Villi
Crypts
blood vessels
DAPI
Epcam
Lgr5
RNApII-pSer2
Merge
e
Insertion points of NPKATPPQI into Venus
M V S K G E E L F T G V V P I L V E L D G D V N G H K F S V S G E G E G D A T Y G
K L T L K L I C T T G K L P V P W P T L V T T L G Y G L Q C F A R Y P D H M K Q H D
F F K S A M P E G Y V Q E R T I F F K D D G N Y K T R A E V K F E G D T L V N R I
E L K G I D F K E D G N I L G H K L E Y N Y N S H N V Y I T A D K Q K N G I K A N F
K I R H N I E D G G V Q L A D H Y Q Q N T P I G D G P V L L P D N H Y L S Y Q S K L
S K D P N E K R D H M V L L E F V T A A G I T L G M D E L Y K P K K K R K V E D A
in vitro (% of wildtype)
CDK9 kinase actvity
in vivo (% of wildtype)
f
Insertion points of NPKATPPQI into mCherry
M V S K G E E D N M A I I K E F M R F K V H M E G S V N G H E F E I E G E G E G R
P Y E G T Q T A K L K V T K G G P L P F A W D I L S P Q F M Y G S K A Y V K H P A
D I P D Y L K L S F P E G F K W E R V M N F E D G G V V T V T Q D S S L Q D G E F
I Y K V K L R G T N F P S D G P V M Q K K T M G W E A S S E R M Y P E D G A L K
G E I K Q R L K L K D G G H Y D A E V K T T Y K A K K P V Q L P G A Y N V N I K L
D I T S H N E D Y T I V E Q Y E R A E G R H S T G G M D E L Y K P K K K R K V E D A
in vitro (% of wildtype)
CDK9 kinase actvity
in vivo (% of wildtype)

Figure S1

Supplementary Figure S1. **RNApII-pSer2 phosphorylation and characterization of CDK9 substrate.**

(a) CDK9 phosphorylates RNApII at multiple Ser2 positions of the RNApII C-terminal YSPTSPS repeats, leading to productive mRNA transcription elongation (simplified presentation). (b) Immunofluorescence of frozen section from mouse brain subventricular zone with the indicated antibodies. (c) Immunofluorescence of frozen section from small intestine of Lgr5-eGFP mice. (d) *In vitro* phosphorylation of bacterially-expressed and purified GST-CTD fusion protein by the indicated cyclin-dependent kinases used as positive control. (e,f) Overview of biochemical properties of all insertion sites of NPKATPPQI into *Venus* and *mCherry*. Amino acid positions are indicated in black above the sequence. Numbers below the amino acid sequence indicate fluorescence percentage (%) of the purified protein with NPKATPPQI inserted at this site compared to wildtype. CDK9 kinase activity towards the peptide insertions is plotted as: - none; + low; ++ medium; +++ high. Constructs with a high CDK9 reactivity were further expressed in mammalian cells (*in vivo*) and their fluorescence compared to wildtype protein. Amino acids in blue are forming loops in the beta barrel structure, in orange is the chromophore, and in red the attached nuclear localization sequence. The orange box indicates the VeN90 insertion position. The optimal insertion position for *Venus* was found at VeN90 because this was the most significant difference between the fluorescence levels in *in vitro* vs *in vivo* cells. The NLS used is indicated at the C-terminus. All the experiments were repeated at least three times with similar results. Source data are provided as a Source Data file.

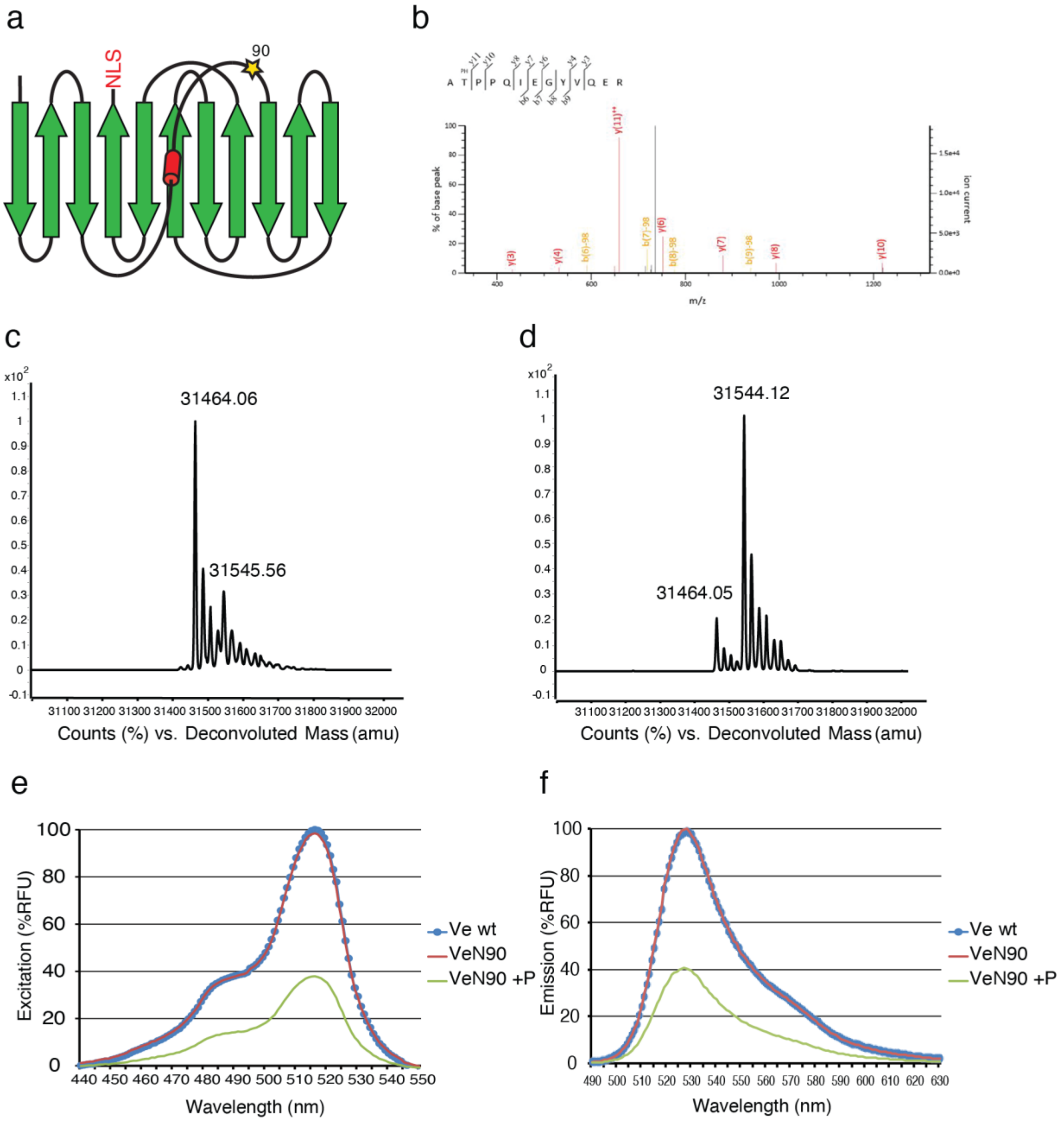

g

Protein	λ_{ex}	λ_{em}	Extinction coeff.	QY	Brightness
Venus	516	528	92200	0.57	52.5
VeN90	516	528	121500	0.55	66.9
VeN90 +P	516	527	47500	0.63	29.8

Figure S2

Supplementary Figure S2. **Characterization of the OSCAR fluorescent reporter.**

(a) Schematic structure of insertion of NPKATPPQI into *Venus*-NLS. Green arrows depict the beta barrel backbone of Venus, the red cylinder shows the chromophore. Note that the kinase substrate was inserted into the first loop after the chromophore in position 90 (aa90), resulting in VeN90. (b) Mass Spec analysis of the purified VeN90 from (d) detects phosphorylation at the expected Threonine NPKAT*PPQI, but no other site. (c) Native qTOF of VeN90 protein purified from Sf9 insect cells detects a mass of 31464 Da, suggesting dehydration (maturation) of the chromophore, N-terminal methionine excision and N-terminal acetylation of the purified protein, two of the most common posttranslational modifications. A minor peak is observed at 31545Da, suggesting a single phosphorylation event in Sf9 insect cells by endogenous kinases. (d) Native qTOF of VeN90 co-expressed in Sf9 insect cells together with CDK9 and CCNT1 and purified results in an 80 Da increase in mass compared to (c), indicative of a single phosphorylation event. (e,f) Absorbance spectrum (e) and emission spectrum (f) of Venus wildtype, unphosphorylated VeN90 and 85% phosphorylated VeN90 (VeN90 +P). (g) Summary of emission and excitation data from (e) and (f).

Figure S3

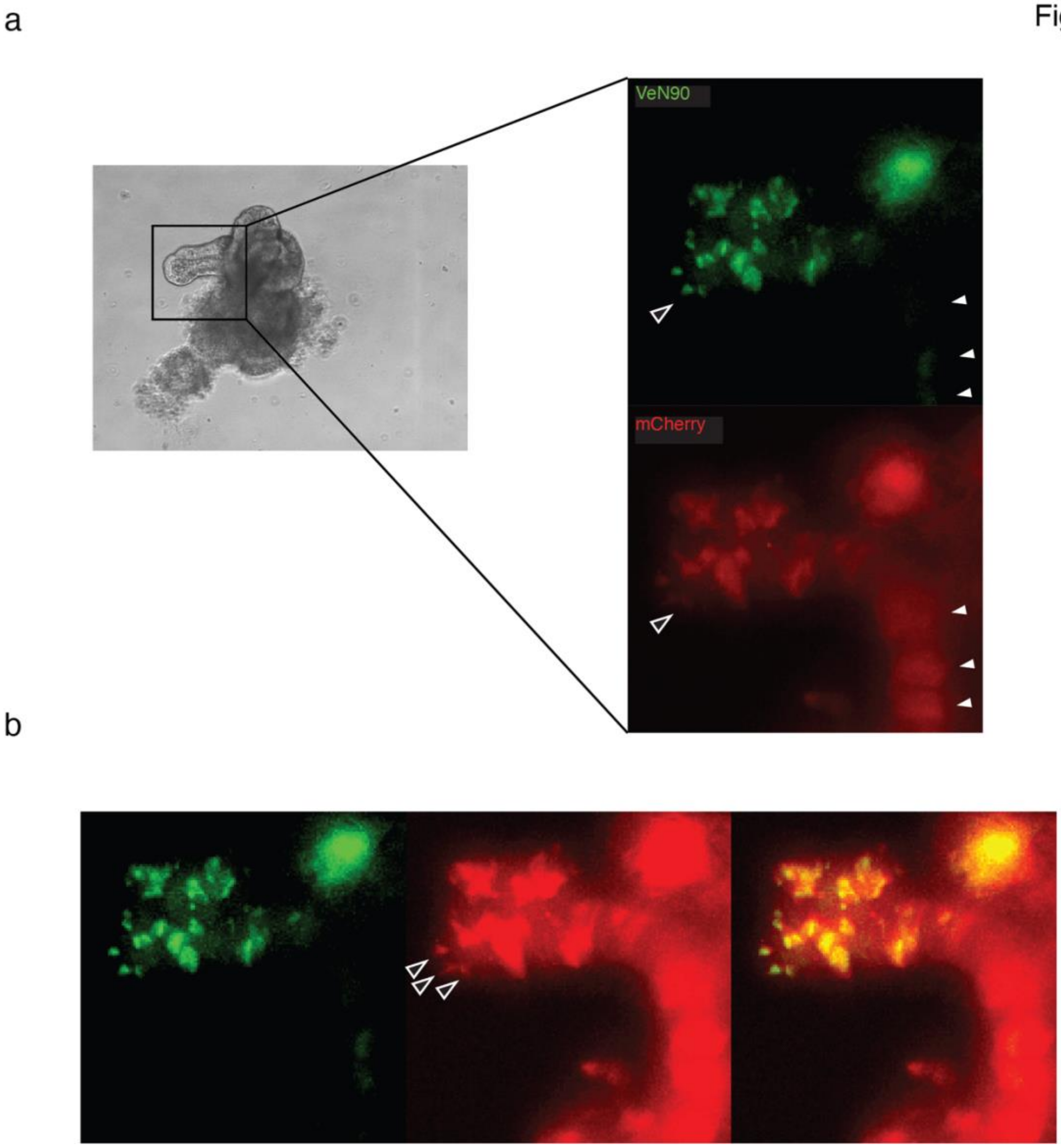

Supplementary Figure S3. Characterization of OSCAR *in vitro*

(a) Enlarged image of the picture shown in Figure 2h. Empty arrowhead indicate cells with high VeN90 / Low mCherry, while filled arrowheads indicate mCherry-positive cells showing low VeN90 fluorescence. (b) Same image as in (a) over-exposed on the mCherry channel. The experiment was repeated at least three times with similar results.

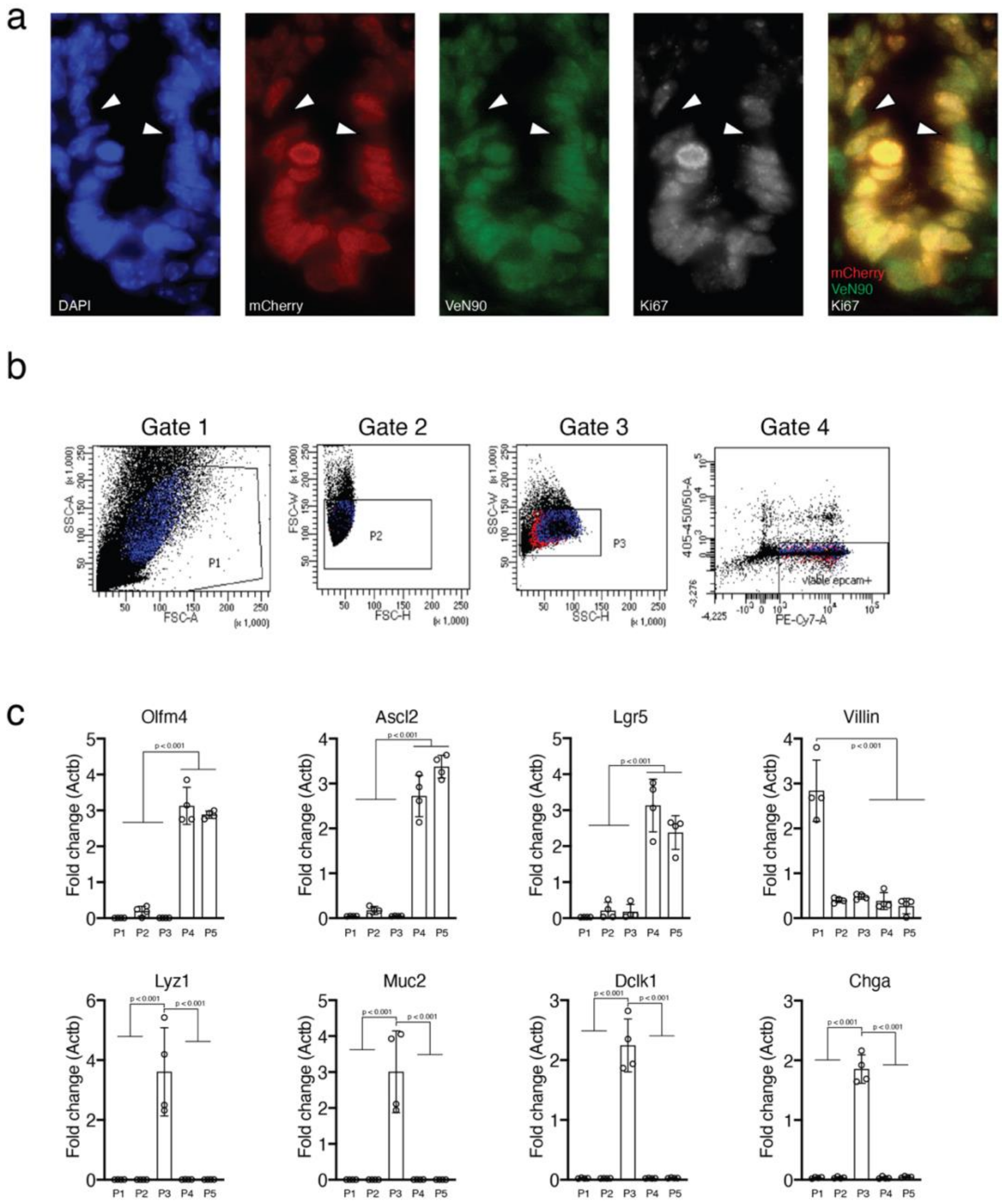

Figure S4

Supplementary Figure S4. Characterization of OSCAR *in vivo*.

(a) Crypts of the small intestine of an EF1a-OSCAR mouse were stained with DAPI and anti-Ki67 antibody. Arrowheads show OSCAR^{high} (VeN90^{high} mCherry^{low}) and Ki67^{low} cells. (b) FACS gating strategy used for analysis of OSCAR fluorescence in intestinal epithelium cells. Cells are filtered for single-cell parameters (gate 2 and 3) and only live Epcam+ (gate 4) cells are analyzed for

OSCAR fluorescence as shown in Figure 4a,b. (c) RT-qPCR analysis of the indicated markers in the indicated OSCAR populations. Ordinary one-way ANOVA test has been used for statistical analysis. Data are presented as mean values +/- SD. (mice = 4). The experiment was repeated at least three times with similar results. Source data are provided as a Source Data file.

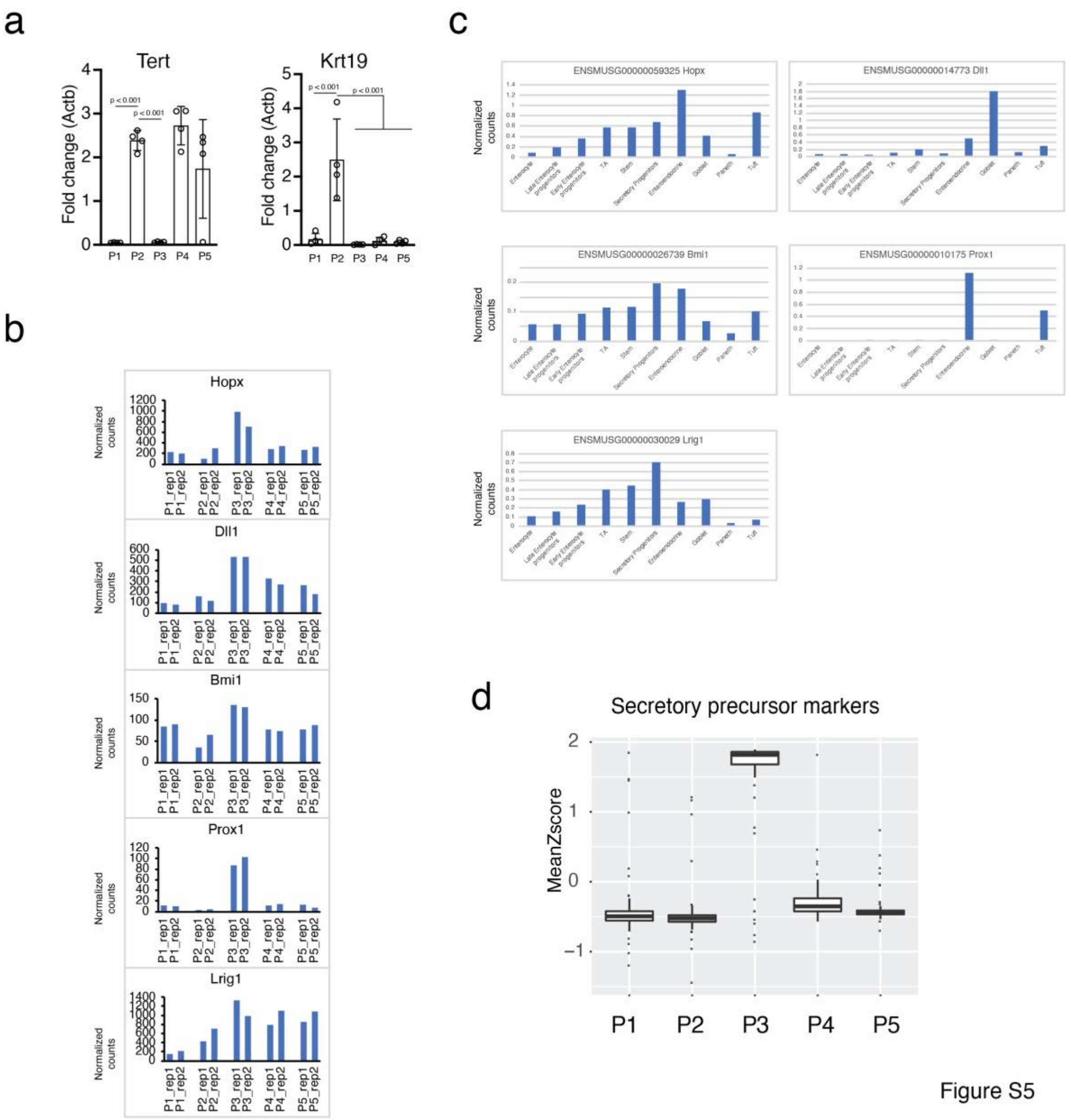

Figure S5

Supplementary Figure S5. Characterization of the different OSCAR populations.

(a) RT-qPCR analysis of the indicated markers in the indicated OSCAR populations. Ordinary one-way ANOVA test has been used for statistical analysis. Data are presented as mean values +/- SD. For Tert gene, P2 vs P1 and P3 vs P2, p value = 0.0002 and for Krt19 gene, P2 vs P1, p value = 0.0002. (mice = 4). (b) Bar charts showing the RNAseq normalized counts levels of the indicated gene markers in the analyzed OSCAR populations. (c) Bar charts showing the single-cell RNAseq normalized counts levels of the indicated gene markers in the different intestinal cell populations. scRNAseq has been in-house generated (data available upon request). (d) Box plot

of geneset enrichment analysis of the secretory precursor markers in the different OSCAR cell populations. P3 cell population contains secretory precursor cells. Boxplots shows the quartile distribution of the data. A distance of 1.5 times the inter quartile range (Q3-Q1) is measured out below the lower quartile and a whisker is drawn up to the lower observed point from the dataset that falls within this distance. All other observed points are plotted as outliers. Source data are provided as a Source Data file.

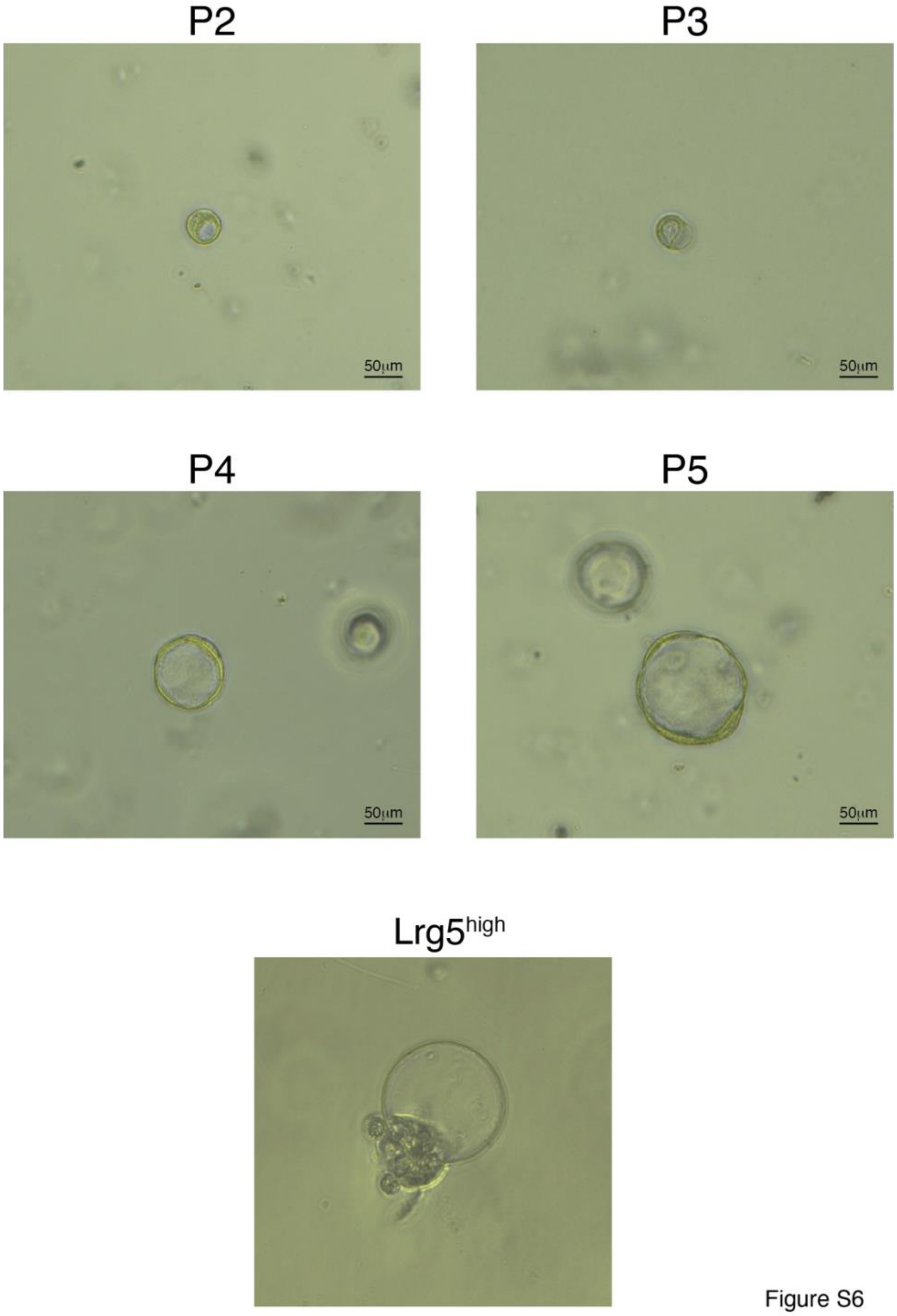

Supplementary Figure S6. Characterization of the different OSCAR populations.

Representative microscopy pictures of single-cell derived cysts after 5 days of culturing of the FACS-sorted intestinal single cells from the different OSCAR cell populations. P2 and P3 populations form cysts but smaller and with lower efficiency and shape with respect to the P4 and P5 populations. Cyst from single Lgr5^{high} cells isolated from Lgr5-eGFP mouse model have been used as control. The experiment was repeated at least three times with similar results.
