# Supplementary material for: Establishment of a fluorescent reporter of RNA-polymerase II activity to identify dormant cells
Source: Nat Commun. 2021 Jun 3;12:3318. doi: 10.1038/s41467-021-23580-4 (PMC8175728; doi:10.1038/s41467-021-23580-4)
Supplement: Supplementary file 2 — Reporting Summary [file 41467_2021_23580_MOESM2_ESM.pdf]

nature research

Corresponding author(s): Francesco Neri

Last updated by author(s): Apr 15, 2021

Reporting Summary

Nature Research wishes to improve the reproducibility of the work that we publish. This form provides structure for consistency and transparency in reporting. For further information on Nature Research policies, see our Editorial Policies and the Editorial Policy Checklist.

Statistics

For all statistical analyses, confirm that the following items are present in the figure legend, table legend, main text, or Methods section.

n/a	Confirmed	
☐	☒	The exact sample size (n) for each experimental group/condition, given as a discrete number and unit of measurement
☐	☒	A statement on whether measurements were taken from distinct samples or whether the same sample was measured repeatedly
☐	☒	The statistical test(s) used AND whether they are one- or two-sided<br>*Only common tests should be described solely by name; describe more complex techniques in the Methods section.*
☒	☐	A description of all covariates tested
☐	☒	A description of any assumptions or corrections, such as tests of normality and adjustment for multiple comparisons
☐	☒	A full description of the statistical parameters including central tendency (e.g. means) or other basic estimates (e.g. regression coefficient) AND variation (e.g. standard deviation) or associated estimates of uncertainty (e.g. confidence intervals)
☐	☒	For null hypothesis testing, the test statistic (e.g. F, t, r) with confidence intervals, effect sizes, degrees of freedom and P value noted<br>*Give P values as exact values whenever suitable.*
☒	☐	For Bayesian analysis, information on the choice of priors and Markov chain Monte Carlo settings
☒	☐	For hierarchical and complex designs, identification of the appropriate level for tests and full reporting of outcomes
☐	☒	Estimates of effect sizes (e.g. Cohen's d, Pearson's r), indicating how they were calculated

Our web collection on statistics for biologists contains articles on many of the points above.

Software and code

Policy information about availability of computer code

Data collection	No software was used
Data analysis	FastQC v0.11.5 : checking the quality of fastq files<br>TopHat v2.1.0 :mapping the sequenced reads to the mm9 genome<br>HTSeq-Count 0.11.2: Counting the number of reads covering each gene<br>R version 3.6.0<br>DESeq2 R package v1.20.0: Calculating p-value and normalized count for RNA-seq data<br>scran R package v1.12.1: Prediction of cell cycle from RNA-seq data<br>Custom script for gene set enrichment analysis (described in the material and method part)<br>DAVID database v6.8: Gene ontology analysis<br>Ingenuity Pathway Analysis (IPA) v45868156: Prediction of potential upstream regulators<br>ImageJ 1.52p: Image analysis<br>Prism 7.0c.:Statistical analysis and plotting

For manuscripts utilizing custom algorithms or software that are central to the research but not yet described in published literature, software must be made available to editors and reviewers. We strongly encourage code deposition in a community repository (e.g. GitHub). See the Nature Research guidelines for submitting code & software for further information.

Data

Policy information about availability of data

All manuscripts must include a data availability statement. This statement should provide the following information, where applicable:

- Accession codes, unique identifiers, or web links for publicly available datasets
- A list of figures that have associated raw data
- A description of any restrictions on data availability

Sequencing data are deposited on GeoDatasets under the Accession Number GSE147030. The reviewer access code is irireocqrjwrluf.

Field-specific reporting

Please select the one below that is the best fit for your research. If you are not sure, read the appropriate sections before making your selection.

[x] Life sciences [ ] Behavioural & social sciences [ ] Ecological, evolutionary & environmental sciences

For a reference copy of the document with all sections, see nature.com/documents/nr-reporting-summary-flat.pdf

Life sciences study design

All studies must disclose on these points even when the disclosure is negative.

Sample size	Sample size was not predetermined. The experiments were done with n > 3 biological replicates, but the RNAseq was done on n = 2. Sample sizes were selected on reaserch-based common standards and on the minimum number of samples allowing statistic (BMJ 2009;338:a3166).
Data exclusions	No data were excluded from the analysis.
Replication	All the experiments were successfully replicated at least 3 times (except RNAseq with only two replicates, but further independently validated by RT-qPCR) also for experiments where only one representative image or result is shown.
Randomization	Biological samples (cells and animals) were randomly selected for the experiments.
Blinding	No blind experiments were performed except for the cyst-forming assay where plating and counting were done by two different persons because of logistic reasons.

Reporting for specific materials, systems and methods

We require information from authors about some types of materials, experimental systems and methods used in many studies. Here, indicate whether each material, system or method listed is relevant to your study. If you are not sure if a list item applies to your research, read the appropriate section before selecting a response.

Materials & experimental systems

n/a	Involved in the study	
[ ]	[x]	Antibodies
[ ]	[x]	Eukaryotic cell lines
[x]	[ ]	Palaeontology and archaeology
[ ]	[x]	Animals and other organisms
[x]	[ ]	Human research participants
[x]	[ ]	Clinical data
[x]	[ ]	Dual use research of concern

Methods

n/a	Involved in the study	
[x]	[ ]	ChIP-seq
[ ]	[x]	Flow cytometry
[x]	[ ]	MRI-based neuroimaging

Antibodies

Antibodies used

Rabbit polyclonal anti-RNA polymerase II CTD repeat YSPTSPS (phospho S2), Abcam, Cat.# ab5095
Mouse monoclonal anti-GFAP antibody (GF5), Abcam, Cat.# ab10062
Rat monoclonal anti-CD45 (30-F11), eBioscience, Cat.# 14-0451-81
Goat polyclonal anti-GFP, Rockland, Cat.# 600-141-215
Goat polyclonal anti-TRP2 (D-18), Santa Cruz, Cat.# sc-10451
Rat monoclonal anti-c-Kit (2B8), APC-conjugated, eBioscience, Cat.# 17-1171-82
Anti-APC microbeads, Miltenyi Biotec, Cat.# 130-090-855
Biotin anti-mouse lineage Panel, BioLegend, Cat.# 133307
Streptavidin APC-Cy7, BioLegend, Cat.# 405208
Rat monoclonal anti-CD34, FITC-conjugated (RAM34), eBioscience, Cat.# 11-0341-82

	Rat monoclonal anti-Sca1, PE-Cy7 conjugated (D7), eBioscience, Cat.# 25-5981-82<br>Rat monoclonal anti-CD150, PE conjugated (9D1), eBioscience, Cat.# 12-1501-82<br>FLAG immunoprecipation kit, Sigma, Cat.# FLAGIPT1-1KT<br>Phos-tag 12.5% gels, Wako, Cat.# 195-17991<br>Mouse monoclonal anti-FLAG (M2), Sigma-Aldrich, Cat.#F1804<br>Rat monoclonal anti-EpCAM (CD326), PE-Cy7 conjugated (G8.8), eBioscience, Cat.# 25-5791-80<br>Rabbit monoclonal antibody Ki67 (SP6) Thermo Scientific, Cat.# RM-9106-S1<br>Alexa Fluor 488 donkey anti-mouse IgG (H+L), Thermo Fisher Scientific, Cat.# A21202<br>Alexa Fluor 568 donkey anti-mouse IgG (H+L), Thermo Fisher Scientific, Cat.# A10037<br>Alexa Fluor 647 donkey anti-mouse IgG (H+L), Thermo Fisher Scientific, Cat.# A31571<br>Alexa Fluor 488 donkey anti-rabbit IgG (H+L), Thermo Fisher Scientific, Cat.# A21206<br>Alexa Fluor 568 donkey anti-rabbit IgG (H+L), Thermo Fisher Scientific, Cat.# A10042<br>Alexa Fluor 647 donkey anti-rabbit IgG (H+L), Thermo Fisher Scientific, Cat.# A31573<br>Alexa Fluor 488 donkey anti-goat IgG (H+L), Thermo Fisher Scientific, Cat.# A11055
Validation	Validation of antibodies was done by the manufacturer.<br>- ab5095: tested applications: ELISA, WB, ICC, IHC-P;<br>species reactivity: Mouse, Rat, Human, Saccharomyces cerevisiae, Recombinant fragment<br>specificity: This antibody recognises the phosphorylated serine found in the amino acid 2 position of the C-terminal domain repeat YSPTSPS.<br>- ab10062: tested applications: IHC-P, WB, ELISA, IHC-FoFr, IHC-Fr, ICC/IF, Flow Cyt, ICC;<br>species reactivity: Mouse, Rat, Human<br>specificity: There is no cross-reactivity with other neurospecific proteins.<br>- 600-141-215: tested applications: WB, ELISA, IF;<br>species reactivity: Tag<br>specificity: No reaction was observed against Human, Mouse or Rat serum proteins.<br>- sc-10451: tested applications: WB, IP;<br>species reactivity: Mouse, Rat, Human, Equine, Canine, Bovine, Porcine<br>specificity: This antibody is raised against a peptide mapping near the N-terminus of TRP2 of human origin.<br>- 17-1171-82: tested applications: WB, IF, IHC, Flow Cyt, AP;<br>species reactivity: Mouse, Human, Pig<br>specificity: The 2B8 monoclonal antibody reacts with mouse CD117, also known as c-Kit receptor.<br>- 133307: tested applications: IF, Flow Cyt;<br>species reactivity: Mouse<br>specificity: The mouse lineage panel has been designed to react with cells from the major hematopoietic cell lineages, such as T lymphocytes, B lymphocytes, monocytes/macrophages, granulocytes, NK cells, and erythrocytes.<br>- 11-0341-82: tested applications: IF, IHC, ICC, Flow Cyt;<br>species reactivity: Mouse, Rat<br>specificity: The RAM34 monoclonal antibody reacts with mouse CD34, also known as mucosialin.<br>- 25-5981-82: tested applications: Flow Cyt;<br>species reactivity: Mouse<br>specificity: The D7 monoclonal antibody reacts with mouse Sca-1, an 18 kDa member of the Ly-6 family of GPI-linked surface proteins. D7 reacts with both Ly-6E.1 and Ly-6A.2 molecules expressed by mouse hematopoietic stem cells, myeloid population and peripheral T and B cells.<br>- 12-1501-82: tested applications: Flow Cyt;<br>species reactivity: Mouse, Fish<br>specificity: The 9D1 monoclonal antibody reacts with mouse CD150, an ~70 kDa transmembrane glycoprotein also known as Signaling Lymphocyte Activation Molecule (SLAM). 9D1 is reported to be an activating antibody.<br>- 25-5791-80: tested applications: Flow Cyt;<br>species reactivity: Mouse, Human<br>specificity: The G8.8 monoclonal antibody reacts with the 40 kDa mouse EpCAM (epithelial cellular adhesion molecule), also known as EGP40 (epithelial glycoprotein 40), 17-1A antigen, TACSTD1 (tumor-associated calcium signal transducer 1), and CD326.<br>- RM-9106-S1: tested applications: Immunohistology<br>species reactivity: Human. Others not tested.<br>specificity: Ki-67

Eukaryotic cell lines

Policy information about cell lines

Cell line source(s)	501mel Human melanoma cell line (female), Obtained from Ruth Halaban, Yale, (Zakut et al, 1993)<br>HEK293T, ATCC, Cat.# CRL-3216<br>Skmel-28 , ATCC, Cat.# HTB-72<br>HeLa, ATCC, Cat.# CCL2
Authentication	None of these lines was additionally in-house authenticated.
Mycoplasma contamination	The used cell lines are mycoplasma free since we do week-based test.

Commonly misidentified lines (See ICLAC register)	No commonly misidentified cell lines were used in the study.

Animals and other organisms

Policy information about studies involving animals; ARRIVE guidelines recommended for reporting animal research

Laboratory animals	Transgenic mouse lines:<br>1) Lgr5-eGFP: B6.129P2-Lgr5tm1(cre/ERT2)Cle/J<br>2) EF1a-lsl-OSCAR: B6.Cg-Gt(ROSA)26Sortm1(EF1a-lsl-OSCAR)<br>3) EF1a-OSCAR: B6.Cg-Gt(ROSA)26Sortm1(EF1a-OSCAR)<br>4) Vasa-Cre: FVB-Tg(Ddx4-cre)1Dcas/J<br><br>Mice used in this study were C57BL6, Lgr5-eGFP, Vasa-Cre or OSCAR mice generated in in-house facility. Mice used were both female and male young adult (4 to 52 weeks old). Generation of the OSCAR mouse line is described in the Methods section.
Wild animals	The study does not involve wild animals.
Field-collected samples	The study does not involve field-collected samples.
Ethics oversight	Thüringer Landesamt für Verbraucherschutz (TLV)

Note that full information on the approval of the study protocol must also be provided in the manuscript.

Flow Cytometry

Plots

Confirm that:

- [x] The axis labels state the marker and fluorochrome used (e.g. CD4-FITC).
- [x] The axis scales are clearly visible. Include numbers along axes only for bottom left plot of group (a 'group' is an analysis of identical markers).
- [x] All plots are contour plots with outliers or pseudocolor plots.
- [x] A numerical value for number of cells or percentage (with statistics) is provided.

Methodology

Sample preparation	For single cells preparation, isolated crypts were dissociated in TrypLE containing Y-27632 (20 µM), Thiazovivin (10 µM) and CHIR99021 (2.5 µM) at 37 °C for 20 minutes. Cells were washed and stained with EpCAM antibody at 1:100 dilution for 30 min on ice.
Instrument	FACS Aria II, FACS ARIA III (BD Biosciences).
Software	FlowJo 10.7.1. PRISM v.8.
Cell population abundance	Quantification of the cells in the different populations showed that the P4 population contains the highest number of cells (~43% of the parental events) followed by the P5 (~16%), P3 (~10%) and finally P1 and P2 (both ~4%) (Figure 4d of the manuscript).
Gating strategy	The FACS gating strategy used in the experiments, especially the FSC/SSC and live/death gates, and boundaries between "positive" and "negative" staining cell populations are described in the figure S4b of the manuscript.

- [x] Tick this box to confirm that a figure exemplifying the gating strategy is provided in the Supplementary Information.
