# Supplementary material for: Establishment of a fluorescent reporter of RNA-polymerase II activity to identify dormant cells
Source: Nat Commun. 2021 Jun 3;12:3318. doi: 10.1038/s41467-021-23580-4 (PMC8175728; doi:10.1038/s41467-021-23580-4)
Supplement: Supplementary file 4 — Description of Additional Supplementary Files [file 41467_2021_23580_MOESM4_ESM.pdf]

Title: Supplementary Movie 1.
Description: Timelapse imaging of mouse small intestinal organoids infected with OSCAR lentivirus. Movie stills are shown in Fig. 2.

Title: Supplementary Data 1.
Description: Raw data of DAPI, mCherry, Ven90 and RNApII-pSer2 (Pol2S2) immunofluorescence signal quantification by using ImageJ. The table also contains OSCAR signal (VeN90/mCherry), RNApII-pSer2 signal normalized on DAPI (Pol2/DAPI) and OSCAR signal normalized on RNApII-pSer2 (OSCAR/Pol2). The quantification comes from 184 (analysis shown in Figure 3), 117, 197, 101 cells coming from 4 independent experiments from 2 different mice. Replicates number 2,3,4 gave the same results as those shown in Figure 3.

Title: Supplementary Data 2.
Description: List of reagents and resources used in this study.
